# Low Concentration of Quercetin Antagonizes the Cytotoxic Effects of Anti-Neoplastic Drugs in Ovarian Cancer

**DOI:** 10.1371/journal.pone.0100314

**Published:** 2014-07-07

**Authors:** Na Li, Chaoyang Sun, Bo Zhou, Hui Xing, Ding Ma, Gang Chen, Danhui Weng

**Affiliations:** 1 Cancer Biology Research Center, Tongji Hospital, Tongji Medical College, Huazhong University of Science and Technology, Wuhan, Hubei, China; 2 Department of Obstetrics and Gynecology, Xiangyang Central Hospital, First Affiliated Hospital of Hubei University of Arts and Science. Xiangyang, Hubei, China; Rajiv Gandhi Centre for Biotechnology, India

## Abstract

**Objective:**

The role of Quercetin in ovarian cancer treatment remains controversial, and the mechanism is unknown. The aim of this study was to investigate the therapeutic effects of Quercetin in combination with Cisplatin and other anti-neoplastic drugs in ovarian cancer cells both *in vitro* and *in vivo*, along with the molecular mechanism of action.

**Methods:**

Quercetin treatment at various concentrations was examined in combination with Cisplatin, taxol, Pirarubicin and 5-Fu in human epithelial ovarian cancer C13* and SKOV3 cells. CCK8 assay and Annexin V assay were for cell viability and apoptosis analysis, immunofluorescence assay, DCFDA staining and realtime PCR were used for reactive oxygen species (ROS)-induced injury detection and endogenous antioxidant enzymes expression. Athymic BALB/c-nu nude mice were injected with C13*cells to obtain a xenograft model for *in vivo* studies. Immunohistochemical analysis was carried out to evaluate the ROS-induced injury and SOD1 activity of xenograft tumors.

**Results:**

Contrary to the pro-apoptotic effect of high concentration (40 µM–100 µM) of Quercetin, low concentrations (5 µM–30 µM) of Quercetin resulted in varying degrees of attenuation of cytotoxicity of Cisplatin treatment when combined with Cisplatin. Similar anti-apoptotic effects were observed when Quercetin was combined with other anti-neoplastic agents: Taxol, Pirarubicin and 5-Fluorouracil (5-Fu). Low concentrations of Quercetin were observed to suppress ROS-induced injury, reduce intracellular ROS level and increase the expression of endogenous antioxidant enzymes, suggesting a ROS-mediated mechanism of attenuating anti-neoplastic drugs. In xenogeneic model, Quercetin led to a substantial reduction of therapeutic efficacy of Cisplatin along with enhancing the endogenous antioxidant enzyme expression and reducing ROS-induced damage in xenograft tumor tissue.

**Conclusion:**

Taken together, these data suggest that Quercetin at low concentrations attenuate the therapeutic effects of Cisplatin and other anti-neoplastic drugs in ovarian cancer cells by reducing ROS damage. Quercetin supplementation during ovarian cancer treatment may detrimentally affect therapeutic response.

## Introduction

Ovarian cancer is the most frequent invasive malignancy of the female genital tract in the United States, with an estimated 22,240 cases diagnosed annually. Approximately 14,030 women die each year from ovarian cancer, representing the most common cause of death among women with gynecological malignancies [1]. Platinum drugs, such as Cisplatin and Carboplatin, are first-line chemotherapeutic agents for the treatment of ovarian cancer. Although most patients display chemosensitivity when beginning therapy, acquired drug resistance has become a major impediment in cancer treatment. The factors that may enhance or suppress the anticancer effect of anti-neoplastic drugs appear to be important in the treatment of ovarian cancer.

Quercetin (3,3′,4′,5,7-pentahydroxyflavone, Quer) belongs to a class of flavonoid compounds, and is in various vegetables, fruits, seeds, nuts, tea, and red wine [2]. It is the major flavonoid in the human diet, with an estimated daily dietary intake of 25 mg in the United States [3]. As a proven antioxidant, Quercetin is recommended to take orally for cancer prevention and health care [4]. In recent years, several studies have noted that Quercetin may act as a potential anticancer drug by enhancing the toxicity of Cisplatin treatment in hepatoma HA22T/VGH and ovarian cancer A2780 cells [5]–[7]. Nevertheless, there are studies reported that in contrast to high concentrations of the flavonoid decreased cell survival and viability, low concentrations increased total antioxidant capacity of cancer cells and prevent cell death due to cytotoxic drugs such as Cisplatin and 5-Fu in lung cancer A549 and colorectal cancer HCT116 cells [8], [9]. The role of Quercetin in ovarian cancer treatment is controversial, and the mechanism of action remains unknown.

Cisplatin and other anti-neoplastic agents lead to increases in intracellular reactive oxygen species (ROS) that may contribute to their therapeutic effect. Antioxidant such as Vitamin C supplements may attenuate the anti-neoplastic activity of drugs that increase ROS [10]. Quercetin is known to reduce intracellular ROS levels in various cell types by promoting the intracellular ROS-scavenging system, which includes modulating detoxifying enzymes, such as superoxide dismutase 1(SOD1) and catalase (CAT). It prompted us to question that whether Quercetin could also negate the cytotoxic effects of anti-neoplastic drugs that increased ROS. The aim of this study was to investigate the effects of Quercetin in combination with Cisplatin and other anti-neoplastic drugs in ovarian cancer cells both *in vitro* and *in vivo*, along with the molecular mechanism of action.

## Materials and Methods

### Ethics Statement

This study was carried out in strict accordance with the recommendations in the Guide for the Care and Use of Laboratory Animals of the National Institutes of Health. The protocol was approved by the Committee on the Ethics of Animal Experiments of Tongji Medical college, Huazhong University of Science and Technology (Permit Number: S292). All efforts were made to minimize suffering of animals.

### Chemicals and reagents

Quercetin (>99% pure), cis-Diammineplatinum(II) dichloride (Cisplatin), Taxol (Paclitaxel) was purchased from Sigma Chemical Co. (St. Louis, MO), dissolved in DMSO, aliquoted, and stored at −20°C. Pirarubicin (Zhejiang Hisun Pharmaceutical Co.,Ltd., China) and 5-Fluorouracil (5-Fu) (Shanghai Xudong Haipu Pharmaceutical Co Ltd., China) were dissolved in normal saline.

### Cell culture

The human epithelial ovarian cancer cell line C13* [11] is the Cisplatin-resistant clone of ov2008 cell line which was derived from a human ovarian carcinoma. This C13* cell line was a gift from Prof. Rakesh at the Ottawa Regional Cancer Center, Ottawa, Canada [3]. SKOV3 ovarian cancer cell line was obtained from American Type Culture Collection (ATCC). Ovarian cancer cells were cultured in RPMI 1640 medium with 10% fetal bovine serum (FBS) at 37°C with 5% CO_2_.

### Cell viability

Cell viability was measured with CCK8 assay (cell counting kit-8, Dojindo Molecular Technologies, Tokyo, Japan). Cells were prepared in 96-well cell culture plates at a cellular density of 5×10^3^ cells/well and treated with Quercetin/anti-neoplastic drugs (Cisplatin, taxol, Pirarubicin and 5-Fu) or vehicle (DMSO with the same dilution rate as the drugs) at 37°C for 48 h. The cell monolayer was washed three times with phosphate-buffered saline (PBS) containing 1.2 mM CaCl_2_ and 0.7 mM MgCl_2_, then a 1∶10 diluted CCK8 solution in RPMI 1640 was added to the cells and incubated for 2 h at 37°C. The results were measured by a microplate reader at 450 nm and expressed as percentages of control values (obtained for cells treated with vehicle).

### Annexin V/PI staining

Cells were trypsinized and washed with serum-containing medium. The samples (5×10^5^ cells) were centrifuged for 5 min at 400×g and the supernatant was discarded. The cells were then stained using an Annexin V-FICT/PI apoptosis kit (keyGEN bioTECH, Nanjing) in accordance with the manufacturer's instructions. The number of apoptotic cells was detected and analyzed using flow cytometry.

### Measurement of ROS

ROS was detected using Reactive Oxygen Species Assay Kit (Beyotime, China) according to manufacturer's instructions. After the molecular probe 5-(and-6)-chloromethyl-2′,7′-dichlorodihydrofluorescein diacetate (DCFH-DA) diffuses into cells and is sequestered intracellularly by de-esterification, the subsequent reaction with peroxides generates fluorescent 5-chloromethyl-2′,7′-dichlorofluorescein (DCF). Briefly, following treatment, cells were collected by centrifugation, resuspended in PBS containing 10 µmol/L DCFH-DA, incubated for 20 min at 37°C, washed with PBS to remove excess dye, and then incubated with RPMI medium at 37°C for 10 min, Fluorescence results were obtained using the FL-1 channel of a Becton Dickinson FACSCalibur, and analyzed using CellQuest Software. The percentage of cells displaying increased dye uptake was used to reflect an increase in ROS levels.

### Immunofluorescence assay

Briefly, C13* cells were seeded in 24-well cell culture plates containing a sterile glass slide at a cellular density of 2×10^4^ cells/well. After treated with different drugs for 48 h, cells were washed three times with ice-cold PBS, and fixed with 4% paraformaldehyde in PBS for 20 min at room temperature. After blocking with 1% non-fat milk for 2 h, goat anti-8-OHdG and mouse anti-γH2AX antibodies (Millipore) were added to the slides respectively. Following 4 h incubation, the slides were washed 3 times with 0.5% PBS-Tween20 (PBST) and the fluorescein CY3-conjugated secondary mouse anti-goat antibody and FITC-conjugated goat anti-mouse secondary antibody (Sigma-Aldrich) were added at a dilution of 1∶100. The cells were incubated for 45 min at 37°C. Finally, the cells were washed as described above and examined with fluorescence microscopy (Olympus, Tokyo, Japan). Five random high power field images were taken of each group, Image J software was used to calculate the numbers of highly positive stained cells.

### Real-time Reverse Transcription Polymerase Chain Reaction (RT-PCR)

C13* cells were seeded in 6-well cell culture plates at a density of 5×10^5^ cells/well, and treated with Quercetin and Cisplatin for 48 h. Total RNA was extracted using Trizol (Invitrogen, China). Complementary DNA was synthesized in accordance with the manufacturers protocol (Toyobo, Japan). Real-time PCR amplification was performed on an ABI PRISM 7500 cycler with SYBR reagent (Toyobo, Japan). The thermal cycling conditions were set as given in the instructions included with the cycler, with an annealing temperature of 60°C. Oligonucleotides used for amplification of Superoxide dismutase 1 (SOD1), endonuclease G (ENDOG), cytochrome c (cyto-c), glutathione peroxidase (GPx), catalase (CAT) and uncoupling protein 2 (UCP2) (Table S1) were designed using Primer 5.0 and synthesized by Invitrogen. Quantitative normalization of cDNA was performed using the housekeeping gene glyceraldehyde-3-phosphate dehydrogenase (GAPDH) as an internal control to determine the uniformity of the template RNA for all specimens. For each sample, the expression of the gene of interest was derived from the ratio of their expression to GAPDH expression using the following formula: relative expression = 2−(ΔCt sample−ΔCt control), ΔCt = Ct gene−Ct GAPDH.

### *In vivo* xenograft studies

The *in vivo* evaluation of Quercetin was carried out using a xenograft model of human C13*cells. Athymic BALB/c-nu nude mice (4–6 weeks old, obtained from Beijing HFK bioscience company, Beijing, China) were housed in a specific pathogen-free room within the animal facilities at the Laboratory Animal Center of University of Tongji Medical College. Animals were allowed to acclimatize to their new environment for one week prior to use. C13* cells (2×10^6^) were resuspended in PBS medium with Matrigel basement membrane matrix (BD Biosciences, Bedford, MA) at a 1∶1 ratio (total volume 100 µL), then were subcutaneously injected into the flanks of nude mice (day 0). From the 10th day of injection, mice were randomly assigned to 4 treatment groups (n = 8 for each group) and injected intraperitoneally (i.p.) with normal saline (NS), Quercetin (20 mg/kg body weight, daily), Cisplatin (4 mg/kg body weight, every four days), and combined Quercetin and Cisplatin treatment (using the above dosages) for 21 consecutive days. Body weight and tumor mass were measured every 5 days. Tumor volume was determined using a caliper and calculated according to the formula (width^2^×length)/2. Mice were killed by cervical dislocation under anesthesia after three weeks treatment (day 30).

### Immunohistochemical analysis

Immunohistochemical studies were performed on the xenograft tumors after they were removed from nude mice. The tumors were fixed in 40 mg/mL paraformaldehyde, paraffin-embedded and cut into 4 µm serial sections. Next, endogenous peroxidases were quenched and the sections were washed carefully with phosphate buffered saline (PBS) three times. The sections were blocked with 2% goat serum and rabbit serum respectively in PBS at 37°C temperature for 45 min, then incubated with mouse anti-SOD1 antibody (1∶500 dilution, Abcam) and goat anti-8-OHdG antibody (1∶800 dilution, Millipore) overnight at 4°C. Afterwards, the sections were incubated with goat anti- mouse and mouse anti-goat horseradish peroxidase-conjugated secondary antibodies separately and avidin-biotin complex followed by diaminobenzidine (Vector ABC, Burlingame, CA, USA). Immersed in 2% ammonia water, haematoxylin was used for counterstaining. Sections were incubated with rabbit and goat IgG serum respectively as negative controls.

Positive 8-OHdG staining was mainly in the nuclei while positive expression of SOD1 was primarily a cytoplasm pattern both in tumor cells rather than in the stromal cells. Because of the intensity of 8-OHdG and SOD1 staining within each section was mostly homogeneous, the immunoreactivity in the samples was semi-quantitatively evaluated using the following criteria: strong positive (scored as 3), strong staining intensity (>90% of cancer cells); moderate positive (scored as 2), moderate staining intensity (>50–89% of positive cells); weak positive (scored as 1), weak staining intensity (>10–49% of positive cells); absent (scored as 0), no staining intensity and no positive or only a few positive cells [12], [13]. The staining intensity and proportion of each section stained were calculated using five random high power field images of each group by two independent investigators.

### Statistical Analysis

All experiments were performed in triplicate. Data are presented as mean ± standard deviation. Statistical analyses were performed using SPSS19.0. Differences between two groups were compared using the Student's *t* test. The statistical differences between more than two groups were determined by one-way ANOVA analysis followed by post hoc pairwise comparisons. *P*<0.05 was considered statistically significant.

## Results

### Quercetin at low concentrations promotes the survival of ovarian cancer C13* cells treated with Cisplatin

**Figure 1:**
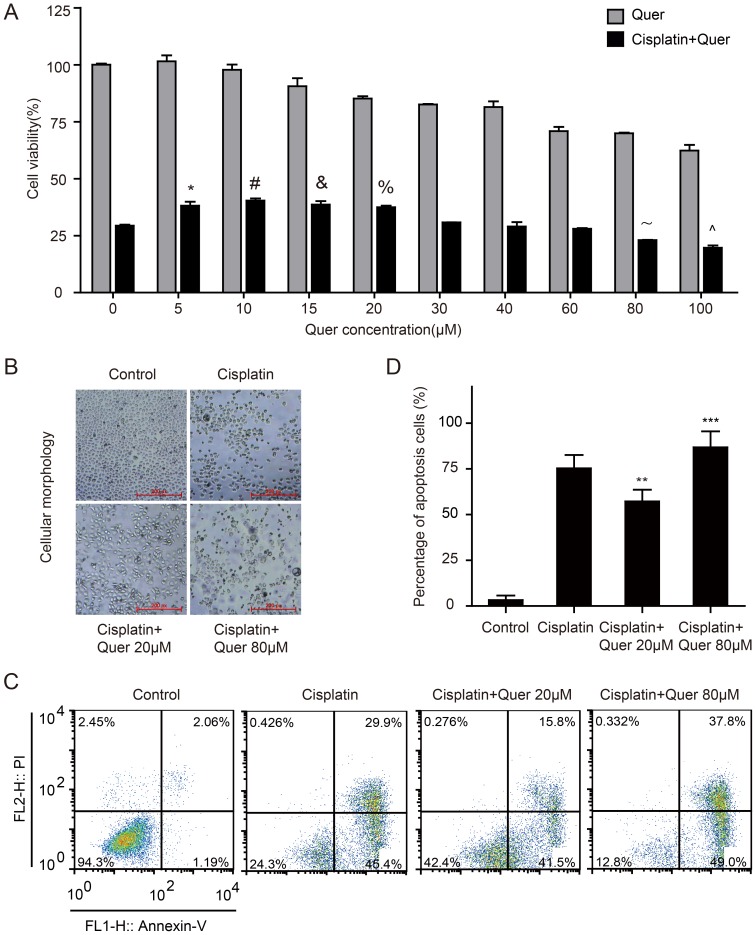
Quercetin in combination with Cisplatin impacts the survival of ovarian cancer C13* cells. (A), Cell viability of groups exposed to different concentrations of Quercetin alone, or combined with 80 µM Cisplatin for 48 hours was measured with CCK8 assay and expressed as percentage of control values. *P = 0.039, #*P* = 0.009, &*P* = 0.027, %*P* = 0.010, ANOVA test followed by post hoc pairwise comparisons. (B), Phase-contrast images of C13* cells treated with vehicle control (DMSO with the same dilution rate as the drugs), Cisplatin, or combination of Quercetin(20 µM,80 µM) with Cisplatin (80 µM). (C, D), Cell apoptosis of different treatment groups was detected and analyzed using flow cytometry. Experiments were performed in triplicate (N = 3 per experiment). Group of Cisplatin combined with Quercetin treatment VS. group of Cisplatin treatment, **P = 0.033, ***P = 0.043, ANOVA test followed by post hoc pairwise comparisons.

Platinum-based chemotherapy is the most important therapy in the treatment of ovarian cancer. To determine the potential role Quercetin may play in Cisplatin resistance in ovarian cancer, C13* cells were exposed to different concentrations of Quercetin, Cisplatin, or a combination of the two. The IC50 of Cisplatin-treated C13* cells was approximately 80 µM (IC50 = 78.8 µM, 95% CI: 72.9–85.1 µM) (Figure S1A). When C13* cells were treated with 80 µM Cisplatin in combination with increasing doses of Quercetin, we found that the cytotoxicity of Cisplatin was reduced when combining with low concentrations of Quercetin (5 µM to 30 µM), while relative high concentration of Quercetin (100 µM) increased Cisplatin cytotoxicity (Fig. 1A). To determine the antagonistic effect or sensitization of the drugs combination, we calculated the combination index (CI) for the drugs based on Chou and Talalay's theorem [14]. The results showed that low concentrations of Quercetin antagonized the cytotoxic effects of cisplatin in C13* cells, while high concentrations of Quercetin have an additive effect with cisplatin (Table S2). We also carried out combination experiments with a series of different cisplatin concentrations plus fixed concentrations of Quercetin (20 µM), which showed that low concentrations of Quercetin increased C13* cell resistance to cisplatin in varying degrees (Figure S2). Phase-contrast images of C13* cells treated with vehicle control, Cisplatin, or combinations of Quercetin (20 µM, 80 µM) with Cisplatin (80 µM) are shown in Fig. 1B.

To further quantify the apoptotic effects of Quercetin and Cisplatin treatment, C13* cells were stained with Annexin-V FITC and PI, and subsequently analyzed by flow cytometry for cell apoptosis. Obvious decreases in numbers of apoptotic cells were detected for cells treated with Cisplatin and 20 µM Quercetin than with Cisplatin alone (Fig. 1C,1D). Proportions of Annexin V-stained cells were higher in Cisplatin-treated cells than those of cells treated with an additional 20 µM Quercetin.

In order to generalize our conclusions, we carried out CCK8 assays in SKOV3, a commonly used ovarian cancer cell line. The results revealed that low concentrations of Quercetin reduced the cytotoxic effects of Cisplatin in SKOV3 cells (Figure S3).

### Quercetin attenuated the therapy effects of different anti-neoplastic drugs in ovarian cancer cells

**Figure 2:**
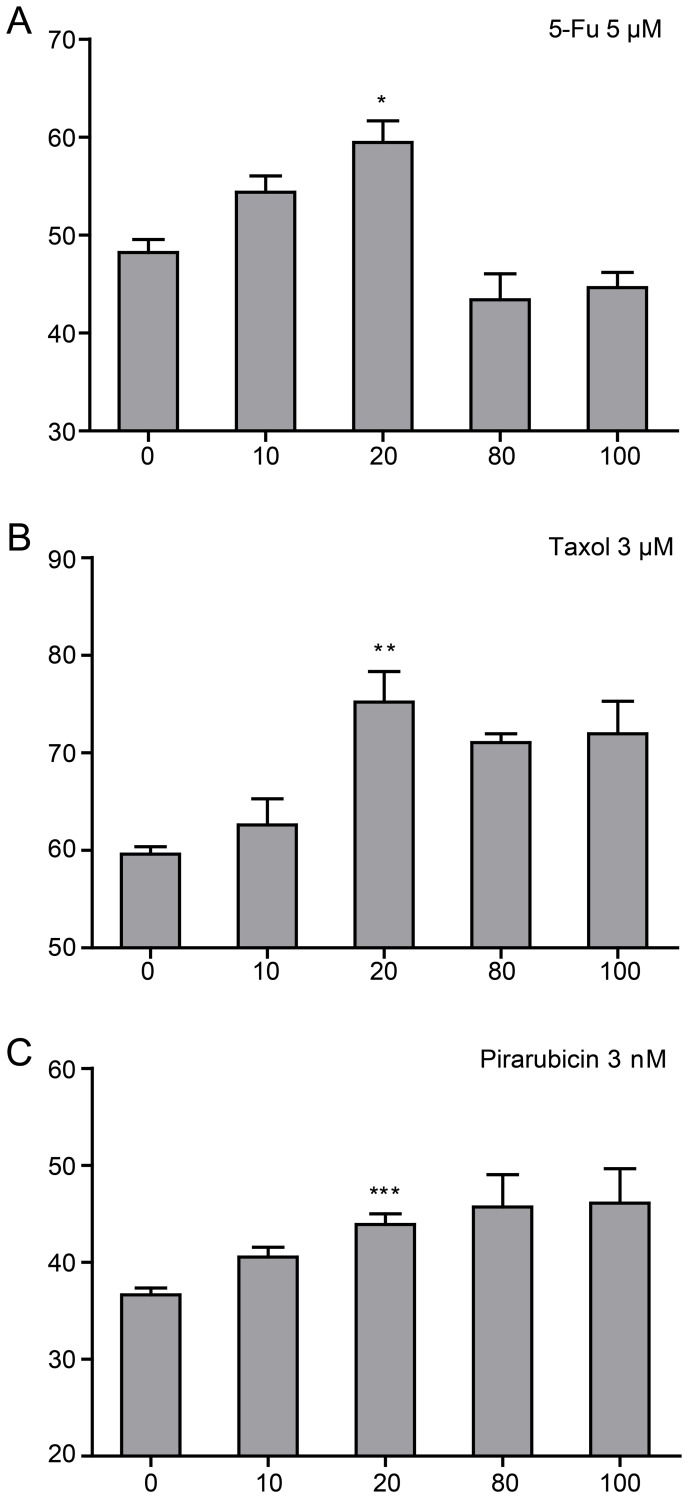
Quercetin in combination with different anti-neoplastic drugs impacts the survival of ovarian cancer C13* cells. (A∼C), Cell viability of Quercetin in combination with 5-Fu, Taxol and Pirarubicin was measured with CCK8 assay and expressed as percentage of control values. N = 3 per experiment. Group of Cisplatin combined with Quercetin 20 µM treatment VS. Group of Cisplatin treatment, *P = 0.048, **P = 0.040, ***P = 0.032, Student t-test.

To examine whether Quercetin attenuate the anti-cancer effects of other anti-neoplastic drugs, we treated C13* cells with three other anti-neoplastic drugs commonly used in ovarian cancer treatment, 5-Fu, Taxol, and Pirarubicin. C13* cells were treated with a series of increasing doses of Quercetin and fixed 5-Fu (5 µM), Taxol (3 µM) and Pirarubicin (3 nM) at approximate concentrations of the IC50 respectively (Figure S1). Similar to the results obtained with Cisplatin, treatment using these drugs in combination with Quercetin resulted in more cell resistance than treatment with anti-neoplastic drugs alone (Fig. 2.) At the concentration of 20 µM, Quercetin showed obvious anti-apoptotic effects against these three drugs. Quercetin showed a low-concentration-specific protecting effect to ovarian cancer cells treated with 5-Fu (Fig. 2A), similar to the results obtained with Cisplatin. In combination with Taxol or Pirarubicin, however, Quercetin maintained the effect of promoting cells survial against anticancer drugs even at relatively high concentrations (80 µM, 100 µM) (Fig. 2B, 2C).

### Quercetin reduced the oxidative injury of ovarian cancer cells caused by Cisplatin

**Figure 3:**
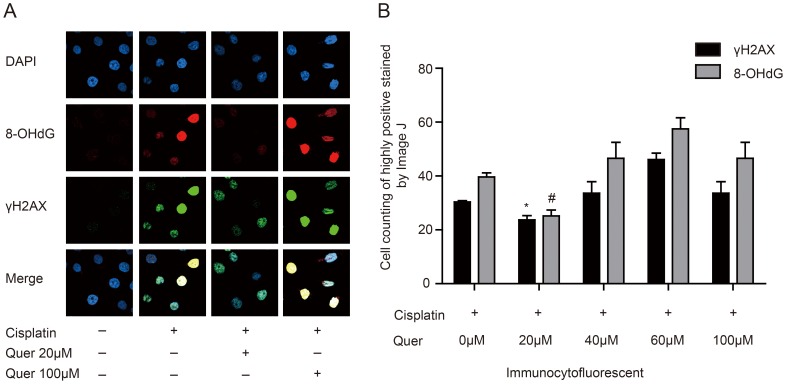
Quercetin reduced oxidative injury of ovarian cancer cells caused by Cisplatin. (A), 8-OHdG and H2AX expression in C13* cells treated with Quercetin in combination with Cisplatin was detected by immunofluorescence assay. (B), Numbers of highly positive stained cells in five random high-power fields were counted by Image J and expressed as percentage of Cisplatin group values. Group of Cisplatin combined with Quercetin 20 µM treatment VS. Group of Cisplatin treatment, N = 3. *P = 0.004, #*P* = 0.001, Student t-test.

We investigated the mechanism by which Quercetin attenuated the cytotoxic effects of Cisplatin. As previously reported [15], many anti-neoplastic drugs including Cisplatin, lead to increases in intracellular ROS that contribute to their therapeutic effect. We questioned whether Quercetin may alter the ROS-associated injury caused by these drugs. The expression of 8-hydroxydeoxyguanosine (8-OHdG), a marker of oxidative DNA stress and the most frequently detected and studied DNA lesion [16], was estimated by immunefluorescence assay. We also measured the level of total cytotoxic injury by detection of γH2AX, a common marker of DNA damage. The results showed that the fluorescence intensities of both γH2AX and 8-OHdG were much lower in the treatment group of 80 µM Cisplatin combined with low concentration Quercetin (20 µM) than the group of 80 µM Cisplatin alone. Contrary to this, 80 µM Cisplatin with high concentration (60 µM, 100 µM) of Quercetin increased the intensity of fluorescence (Fig. 3A, 3B). Counting of positive cells using Image J software showed that combination treatment with Quercetin (20 µM) had a more obvious decrease of 8-OHdG than that of γH2AX, compared to Cisplatin treatment alone (36.40% and 19.33% respectively) (Fig. 3B). It indicated that low concentrations of Quercetin reduced the oxidative injury of ovarian cancer cells caused by Cisplatin.

### Quercetin reduced ROS level of ovarian cancer cells undergoing Cisplatin treatment

**Figure 4:**
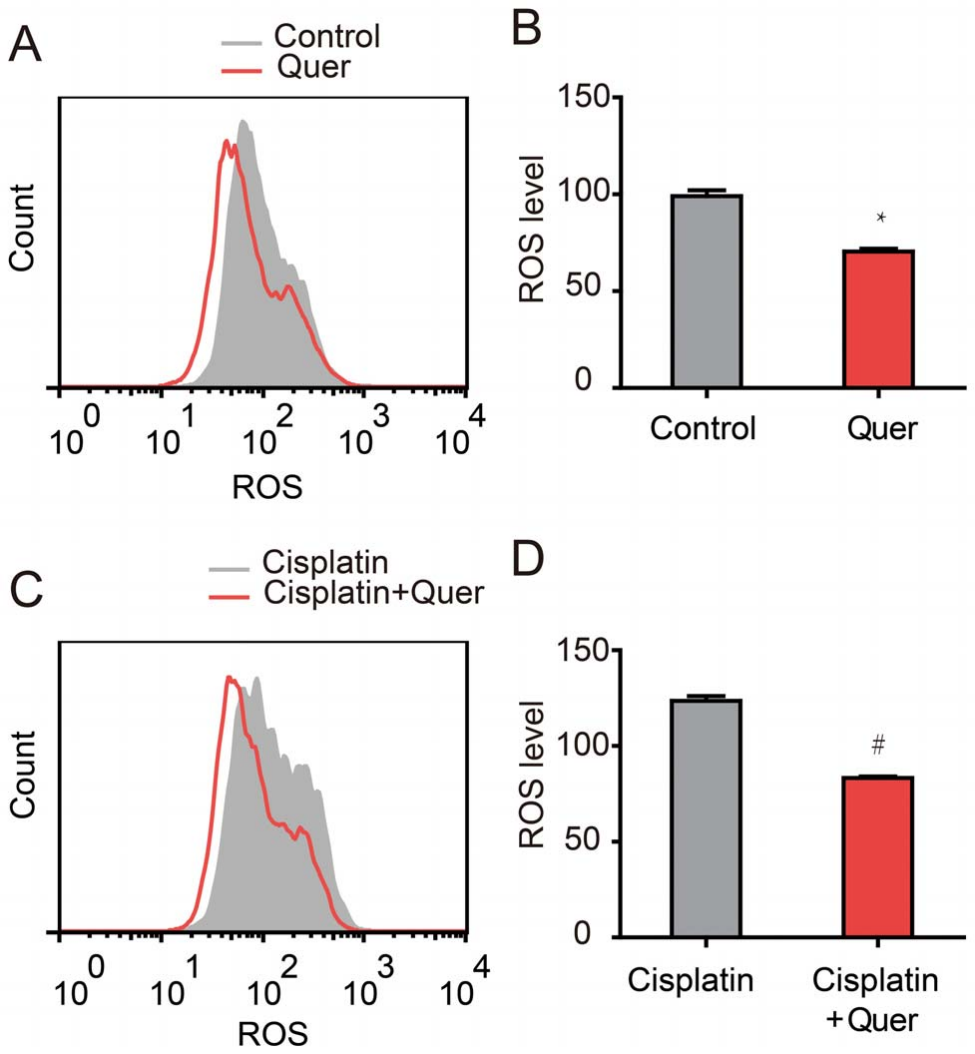
Quercetin reduced ROS level of ovarian cancer cells in combination with Cisplatin treatment. Intracellular ROS levels of C13* cells of different treatment groups were detected by Reactive Oxygen Species Assay using flow cytometry. (A, B) The ROS levels of C13* cells treated by vehicle control or 20 µM Quercetin for 24 hours. (C, D) The ROS levels of C13* cells treated by 80 µM Cisplatin with or without 20 µM Quercetin for 24 hours. N = 3. *P<0.001, #*P*<0.001, Student t-test.

To determine whether Quercetin reduced the ROS injury by decreasing intracellular ROS, Reactive Oxygen Species Assay Kit was used to detect intracellular ROS levels of C13* cells in different treatment groups. Compared to cells treated with vehicle control or Cisplatin alone, treatment with an additional 20 µM Quercetin lead to a reduction in intracellular levels of ROS (Fig. 4A, 4C). Quantitative analysis showed that Quercetin (20 µM) treatment gave an obvious reduction in ROS level (mean value 70.4) compared to the control group (mean value 99.05) (*P*<0.001) (Fig. 4B, 4D). In agreement with previous results, exposure to Cisplatin caused ovarian cancer C13* cells to accumulate the intracellular ROS, with a mean ROS level of 123.5. In treatment group of Cisplatin combined with Quercetin, this value was reduced to 83.38 (Fig. 4D), even lower than the vehicle control group (99.05) (*P*<0.001) (Fig. 4B).

### Quercetin increased the expression of endogenous antioxidant enzymes in ovarian cancer cells

**Figure 5:**
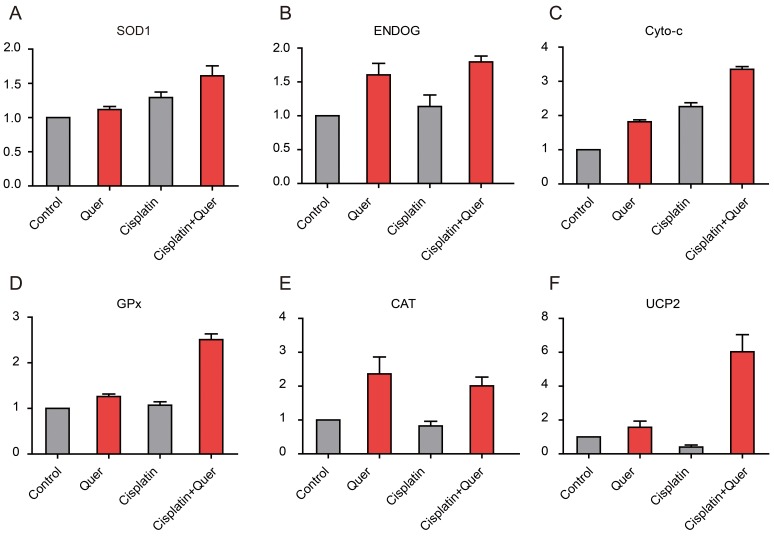
Quercetin increased the expression of endogenous antioxidant enzymes in ovarian cancer cells. (A∼F), Expression of antioxidant enzymes of C13* cells was detected by real-time PCR. N = 3.

The results above indicated that Quercetin could reduce intracellular ROS of ovarian cancer cell, so we sought to determine a mechanism of action. The most important antioxidant components of cells against ROS are the endogenous antioxidant enzymes, including Superoxide dismutase 1 (SOD1), endonuclease G (ENDOG), cytochrome c (cyto-c), glutathione peroxidase (GPx), catalase (CAT), and uncoupling protein 2 (UCP2). We measured the expression of endogenous antioxidant enzymes by realtime PCR. Compared to cells treated with vehicle control or Cisplatin alone, treatment combining Cisplatin with 20 µM Quercetin lead to various degrees of increase in the expression of SOD1, ENDOG, cyto-c, GPx, CAT and UCP2 (Fig. 5A∼F).

### Quercetin promoted ovarian cancer growth with Cisplatin treatment *in vivo*

**Figure 6:**
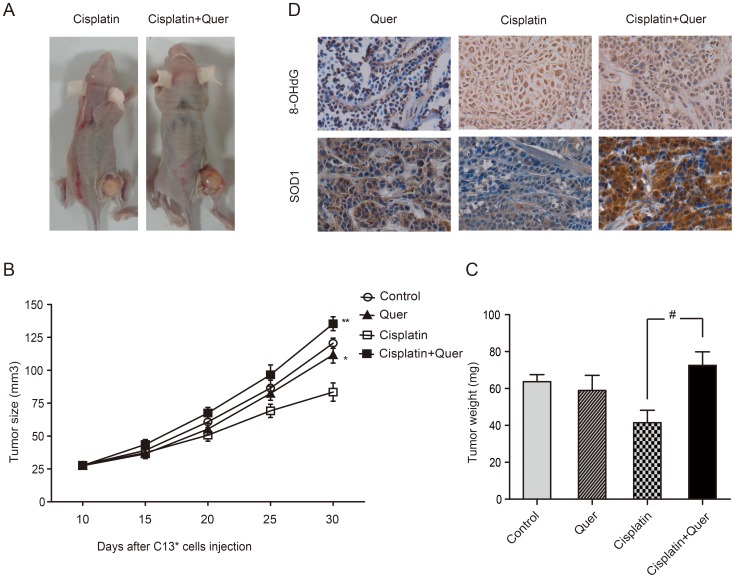
Quercetin in combination with Cisplatin promoted ovarian tumor growth. Mice were injected with NS, 20/kg Quercetin, 4 mg/kg Cisplatin, or Quercetin plus Cisplatin, ip. C13* cell xenografts (2×10^6^) were inoculated in the right flank of female athymic nude nu/nu mice. Tumor volumes was determined by a caliper and calculated according to the formula (width2×length)/2. (A), Tumor formation in mice of Cisplatin treatment group and combined with Quercetin group. (B), Average relative tumor growth as analyzed by the increase in tumor volumes for 8 mice per experimental group **Cisplatin plus Quercetin VS. Cisplatin, *P*<0.001; *Quercetin VS. Control, *P* = 0.015, ANOVA test followed by post hoc pairwise comparisons; (C), Average tumor weights of each group. #Cisplatin combined with Quercetin VS. Cisplatin, *P*<0.001, ANOVA test followed by post hoc pairwise comparisons; (D), Immunohistochemical analysis of 8-OHdG and SOD1 in xenograft tumors removed from nude mice. (E), The quantification data of the differences in the expression SOD-1 and 8-OHdG, *: P<0.001, **:P<0.001, ANOVA test followed by post hoc pairwise comparisons.

We used C13* cells to generate xenograft tumors in athymic nude BALB/c-nu mice to determine whether Quercetin could attenuate the effects of chemotherapy *in vivo*. As expected, treatment with Cisplatin alone reduced tumor growth compared with vehicle treated mice. Treatment with Quercetin alone slightly suppressed tumor growth compared with normal saline control (tumor volumes of 111.75 mm^3^ and 120.51 mm^3^ respectively) (P = 0.015). Tumors from mice treated with Cisplatin in combination with Quercetin were approximately 1.8 times larger at day 30 than those treated with Cisplatin alone (*P*<0.001; Fig. 6A, 6B). Comparing the average weights of tumors removed from mice, we found that tumors treated with combination of Quercetin and Cisplatin (72.53±7.33 mg) were significantly heavier than that treated with Cisplatin alone (41.47±6.72 mg), *P*<0.001 (Fig. 4D). Further, the combination of Cisplatin and Quercetin treatment displayed a cancer promoting effect compared to the control group, measured both in tumor size and in tumor weight (Fig. 6C).

### Quercetin enhanced the expression of endogenous antioxidant enzyme SOD1 of ovarian cancer cells *in vivo*, and prevented ROS-induced damage

In order to confirm the protective effect against oxidative injury by Quercetin in vivo, immunohistochemical assays were carried out in tumors removed from nude mice xenograft model. 8-OHdG, a marker of oxidative DNA stress, was located mainly in cancer cell nucleus. In tumors treated with vehical or Quercetin alone, 8-OHdG staining showed weak intensity with the scores of 0.4 and 0.6 respectively. In the group treated with Cisplatin alone, almost all cancer cells displayed extremely strong 8-OHdG staining with score 2.8. As expected, mice group treated by Cisplatin in combination with Quercetin had a much lower level of 8-OHdG staining (score 1.6) in tumors than the one treated with Cisplatin alone (Fig. 6D–E).

DNA repair enzymes prevent the accumulation of damaged DNA and antioxidants protect cells against free radicals. We detected SOD1 expression, which is a critical endogenous antioxidant enzyme for tolerating the oxidative stress, to find out if Quercetin affected on the activity of endogenous antioxidant enzyme. Positive expression of SOD1 was primarily a cytoplasm pattern in ovarian cancer cells rather than stromal cells. The antioxidant enzyme SOD1 was highly overexpressed in tumors treated with Quercetin or Cisplatin plus Quercetin (score 2.0 and 2.6 respectively) compared to tumors treated with vehicle or Cisplatin alone (score 1.2 and 0.6 respectively) (Fig. 6D–E). The results indicated that Quercetin enhanced the endogenous antioxidant enzyme SOD1 expression of ovarian cancer cells *in vivo*, and prevented ROS-induced damage.

## Discussion

As a classical flavonoid compound, Quercetin is usually recommended to take orally every day for general health care and cancer prevention. Staedler *et al.* found that Quercetin at concentrations of 5 µM and 10 µM could increase the efficacy of 100 µM doxorubicin in breast cancer cells in vitro [17], [18], while other researchers came to different conclusions [8], [9]. Samuel *et al.* [8] reported that in colorectal and prostate cancer cells, the therapeutic effects of drug in combination with Quercetin were influenced by the effective doses and the p53 status of the cells (the combination of 5-Fu with up to 6 µM Quercetin promoted cologenic survival in p53 null cells while 50 µM Quercetin acted opposite role in p53 wild type cells). In this study, Quercetin at high concentrations, either alone or combined with Cisplatin displayed anti-neoplastic effects in ovarian cancer C13* cells, while low concentration (5 µM–30 µM) of Quercetin appeared to antagonize the cytotoxic effects of anti-neoplastic agents including Cisplatin, 5-Fu, Taxol, and Pirarubicin. We also explored the mechanism of the anti-apoptotic effect of Quercetin when combined with Cisplatin. C13* cells treated with additional 20 µM Quercetin showed a decreased intracellular ROS levels and elevated expression of endogenous antioxidant enzymes including SOD1, ENDOG, cyto-c, GPx, CAT, and UCP2, than cells treated with Cisplatin alone. In a nude mouse xenograft model injected with C13* cells, daily intraperitoneal injection of Quercetin at 40 mg/kg led to a substantial reduction of therapeutic efficacy of Cisplatin. Tumors in mice treated with Cisplatin in combination with Quercetin displayed enhanced SOD1 expression and decreased 8-OHdG staining than those of mice treated with Cisplatin alone. Overall, our data indicated that administration of Quercetin at low concentrations may antagonize the cytotoxic effects of anti-neoplastic drugs in ovarian cancer cells, and that Quercetin decreased the levels of oxidative injury caused by Cisplatin.

Researchers have not reached a unanimous conclusion about the absorption of Quercetin intake, and taking into account individual differences in absorption and different dosage forms, the plasma concentration after a certain dose of Quercetin taken was not under control. It has been reported that a 100 mg single dose Quercetin taken orally was found to result in a serum concentration of 0.8 mM [19], while aother report showed that a 4 g oral dose of Quercetin led to no measurable Quercetin in either the plasma or urine of healthy volunteers [20]. A recent study showed that, in healthy volunteers supplemented with 50, 100, or 150 mg/day Quercetin orally for 2 weeks, plasma concentrations of Quercetin were 145 nmol/L, 217 nmol/L and 380 nmol/L respectively [21], which were all lower than the low-concentration dosage (20 µM) used for our *in vitro* experiments. It's hard to control the exact plasma concentration of Quercetin for individuals to intake it. As the results of the present study, low concentration of Quercetin may attenuate the therapeutic effects of Cisplatin and some other anti-neoplastic drugs, so Quercetin supplementation in ovarian cancer patients during chemotherapy may be antagonistic to the cytotoxic effects of chemotherapy.

## Supporting Information

Figure S1 The IC50 values of C13* cells treated with four anti-neoplastic drugs respectively. Cell viability of C13* cells exposed to a series of concentrations of Cisplatin, 5-Fu, Taxol and Pirarubicin for 48 hours was measured using CCK8 assay and expressed as percentage of control values (A∼D).
(TIF)

Figure S2 Quercetin at a low concentration (20 µM) improved C13* cells survival in combination of different concentrations of Cisplatin treatment.
(TIF)

Figure S3 The IC50 value and cell viability of SKOV3 cells exposed to a series of concentrations of Cisplatin for 48 hours was measured using CCK8 assay (A); Cell viability of of SKOV3 cells exposed to different concentrations of Quercetin alone, or combined with 50 µM Cisplatin for 48 hours was measured using CCK8 assay and expressed as percentage of control values (B).
(TIF)

Table S1 Primers used in this study for real-time PCR experiments.
(DOC)

Table S2 The combination indexs of different concentration of Quercetin in combinations with Cisplatin.
(DOC)
