# Supplementary figures and images for: Low Concentration of Quercetin Antagonizes the Cytotoxic Effects of Anti-Neoplastic Drugs in Ovarian Cancer
Source: PLoS One. 2014 Jul 7;9(7):e100314. doi: 10.1371/journal.pone.0100314 (PMC4085066; doi:10.1371/journal.pone.0100314)

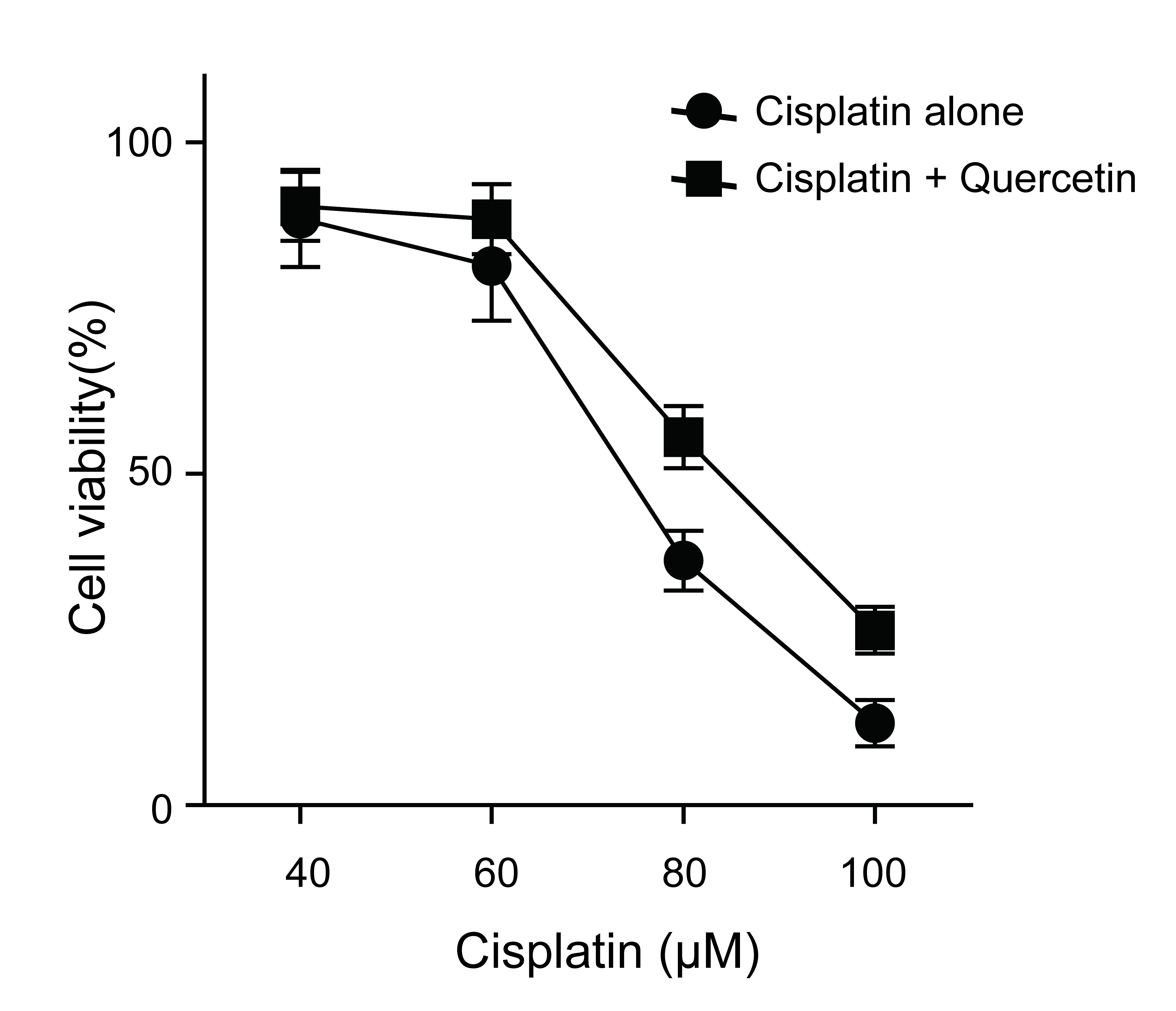

Supplement: Figure S2 — Quercetin at a low concentration (20 µM) improved C13* cells survival in combination of different concentrations of Cisplatin treatment. (TIF) [file pone.0100314.s002.tif]

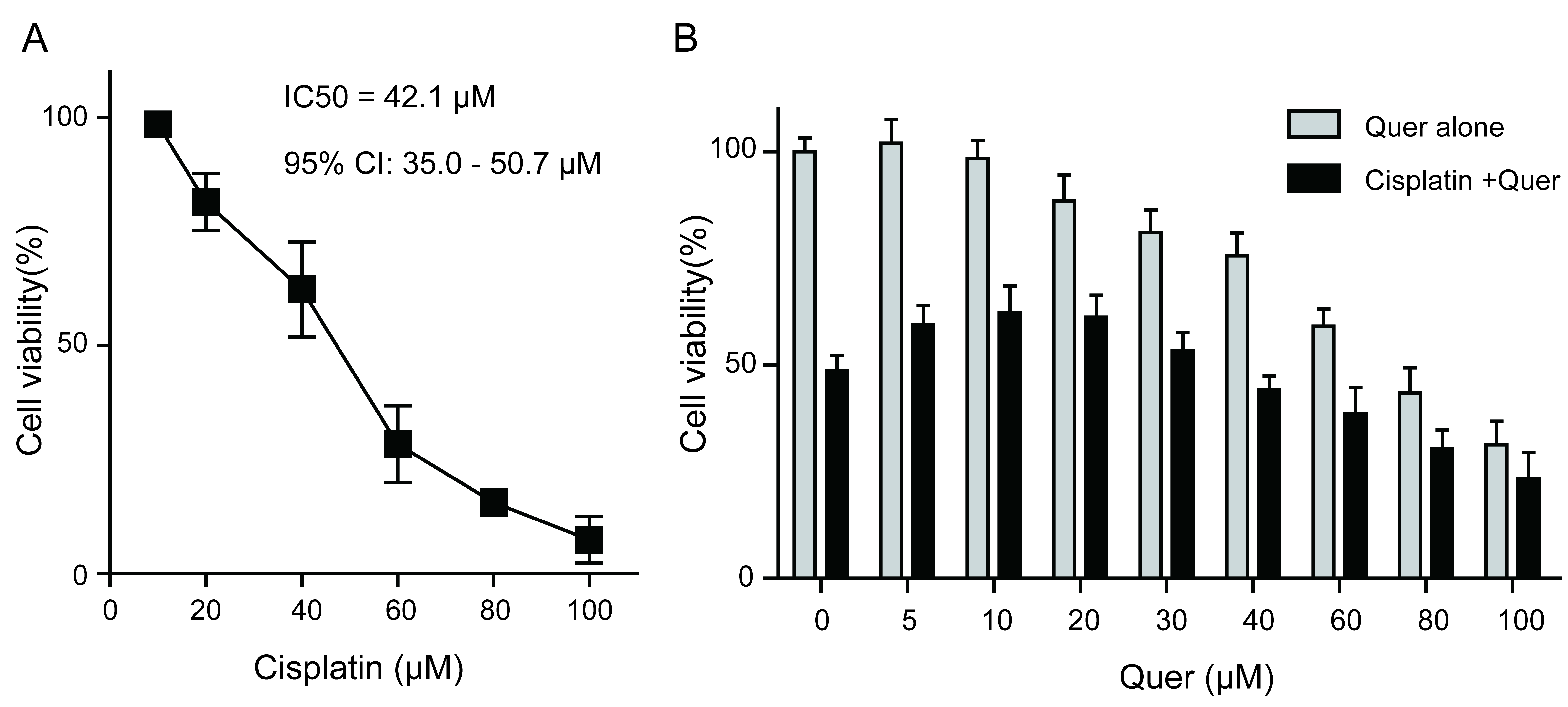

Supplement: Figure S3 — The IC50 value and cell viability of SKOV3 cells exposed to a series of concentrations of Cisplatin for 48 hours was measured using CCK8 assay (A); Cell viability of of SKOV3 cells exposed to different concentrations of Quercetin alone, or combined with 50 µM Cisplatin for 48 hours was measured using CCK8 assay and expressed as percentage of control values (B). (TIF) [file pone.0100314.s003.tif]
