# Supplementary material for: Low Concentration of Quercetin Antagonizes the Cytotoxic Effects of Anti-Neoplastic Drugs in Ovarian Cancer
Source: PLoS One. 2014 Jul 7;9(7):e100314. doi: 10.1371/journal.pone.0100314 (PMC4085066; doi:10.1371/journal.pone.0100314)
Supplement: Table S1 — Primers used in this study for real-time PCR experiments. (DOC) [file pone.0100314.s004.doc]

Supplementary Table1. Primers used in this study for real-time PCR experiments.

| Name | Primer | Sequence (5'->3') | Tm |
| --- | --- | --- | --- |
| SOD1 | Forward primer | TGGTTTGCGTCGTAGTCTCC | 60.04 |
| SOD1 | Reverse primer | CCAAGTCTCCAACATGCCTCT | 60.00 |
| ENDOG | Forward primer | AGTCGTACGTGCTGTGCTAC | 60.11 |
| ENDOG | Reverse primer | CATCAGCCTCTGTCCTGGG | 59.47 |
| cyto-c | Forward primer | CGGGGTGCCTTTAGGATTCA | 55.00 |
| cyto-c | Reverse primer | TTCTGACAGCGGTGGAAGTC | 55.00 |
| GPx | Forward primer | GTCGGTGTATGCCTTCTCGG | 59.61 |
| GPx | Reverse primer | TCTTGGCGTTCTCCTGATGC | 60.39 |
| CAT | Forward primer | ACTGTTGCTGGAGAATCGGG | 60.04 |
| CAT | Reverse primer | AGGACGTAGGCTCCAGAAGT | 59.96 |
| UCP2 | Forward primer | AGCCCACGGATGTGGTAAAG | 60.04 |
| UCP2 | Reverse primer | AGCCCACGGATGTGGTAAAG | 60.11 |
| GAPDH | Forward primer | GAAAGCCTGCCGGTGACTAA | 60.32 |
| GAPDH | Reverse primer | GATCTCGCTCCTGGAAGATGG | 60.00 |
