# Supplementary material for: Low Concentration of Quercetin Antagonizes the Cytotoxic Effects of Anti-Neoplastic Drugs in Ovarian Cancer
Source: PLoS One. 2014 Jul 7;9(7):e100314. doi: 10.1371/journal.pone.0100314 (PMC4085066; doi:10.1371/journal.pone.0100314)
Supplement: Table S2 — The combination indexs of different concentration of Quercetin in combinations with Cisplatin. (DOC) [file pone.0100314.s005.doc]

Supplementary Table 2: The combination indexs of different concentration of Quercetin in combinations with Cisplatin

| Dose of Quercetin(μM) | Dose of Cisplatin(μM) | CI |
| --- | --- | --- |
| 5.0 | 80.0 | 1.11103 |
| 10.0 | 80.0 | 1.28347 |
| 15.0 | 80.0 | 1.21250 |
| 20.0 | 80.0 | 1.15834 |
| 30.0 | 80.0 | 1.1513 |
| 40.0 | 80.0 | 0.97436 |
| 60.0 | 80.0 | 0.96448 |
| 80.0 | 80.0 | 0.96916 |

CI: combination index. The combination index (CI) theorem of Chou-Talalay offers quantitative definition for additive effect (CI = 1), synergism (CI < 1), and antagonism (CI > 1) in drug combinations. The CI value were calculated with CompuSyn software developed based on Chou and Talaly' algorithm.

1. Chou TC**: Theoretical basis, experimental design, and computerized simulation of synergism and antagonism in drug combination studi**es*. Pharmacological review*s 2006**,** 58(3):621-681.
